# Vitamin D, the Vitamin D Receptor, Calcitriol Analogues and Their Link with Ocular Diseases

**DOI:** 10.3390/nu14112353

**Published:** 2022-06-05

**Authors:** Miłosz Caban, Urszula Lewandowska

**Affiliations:** Department of Biochemistry, Faculty of Medicine, Medical University of Lodz, Kosciuszki 4, 90-419 Lodz, Poland; milosz.caban@stud.umed.lodz.pl

**Keywords:** 25-hydroxyvitamin D, calcitriol, eye diseases, vitamin D

## Abstract

The global prevalence of eye diseases continues to grow, bringing with it a reduction in the activity levels and quality of life of patients, and partial or complete blindness if left untreated. As such, there is considerable interest in identifying more effective therapeutic options and preventive agents. One such agent is vitamin D, known to have a range of anti-cancer, anti-angiogenic, anti-inflammatory and anti-oxidative properties, and whose deficiency is linked to the pathogenesis of a range of cardiovascular, cancer, and inflammatory diseases. This review presents the current stage of knowledge concerning the link between vitamin D and its receptor and the occurrence of eye disease, as well as the influence of analogues of calcitriol, an active metabolite of vitamin D. Generally, patients affected by various ocular disorders have vitamin D deficiency. In addition, previous findings suggest that vitamin D modulates the course of eye diseases and may serve as a marker, and that its supplementation could mitigate some disorders. However, as these studies have some limitations, we recommend further randomized trials to clarify the link between vitamin D and its activity with eye disease.

## 1. Introduction

The human eye is a delicate structure that receives optical information from the environment, allowing light perception and vision. However, its operation is inhibited by a range of factors, such as aging, genetic predisposition, excessive light exposure, chronic hyperglycemia, autoimmune diseases, angiogenesis, inflammation and oxidative stress; these may lead to the development of a range of eye diseases such as age-related macular degeneration, diabetic retinopathy or dry eye syndrome. In addition, most ocular disorders result in visual impairment or blindness. Approximately 405 million individual cases of visual impairment were reported in 2015, with 36 million people living with vision loss that year. Moreover, the number of patients with blindness is expected to increase about threefold by the year 2050 [1]. Visual impairment is also related to considerable economic burden for patients, their caregivers and medical care in developed countries [2]. In addition, blindness and visual impairment are two of the strongest risk factors for social isolation, reduced quality of life and decline of functional status. Furthermore, the majority of ocular diseases are diagnosed in advanced stages, which excludes effective therapy [3,4]. As such, prevention is a priority and a significant element of therapeutic approaches.

Numerous studies have demonstrated that a healthy diet limits the development or progression of eye disorders. Indeed, vitamin D deficiency, i.e., a 25-hydroxyvitamin D level lower than 20 ng/mL, is a global health problem [5]. The consumption of fruits and vegetables, which are source of polyphenols and carotenoids, as well as vitamins A, C and E, play a significant role in the prevention and treatment of ocular diseases. These compounds have multiple pro-health properties, mainly comprising anti-inflammatory, anti-oxidant and anti-angiogenic activities [6,7,8]. Nevertheless, other agents believed to more effectively inhibit the development of eye diseases have been the focus of many studies. One such agent is vitamin D, commonly defined as the “sunshine vitamin”. Although it is a multifunctional hormone, its primary purposes are arguably to maintain calcium homeostasis, mineral metabolism and skeletal health. Vitamin D binds calcitriol, an activated metabolite of vitamin D, to the vitamin D receptor (VDR). Interestingly, recent studies indicate that vitamin D and calcitriol derivatives may mitigate the course of many diseases inter alia cancers, psoriasis, multiple sclerosis, diabetes and autoimmune diseases [9,10,11]. In addition, increasing numbers of studies show that vitamin D, VDR and calcitriol analogues can play an important role in the maintenance of ocular health [12]. This aim of this paper is to review existing literature regarding the role of vitamin D, VDR and calcitriol analogues in eye diseases.

## 2. Vitamin D—Sources, Metabolism and VDR

In humans, vitamin D can be obtained from three potential sources: food, local synthesis in the skin and supplementation. It is available in two major forms: cholecalciferol (vitamin D_3_) and ergocalciferol (vitamin D_2_). The main dietary sources are fatty fishes and fish oils, primarily mackerel, sardines, salmon and cod liver oil in the case of vitamin D_3_, and sundried mushrooms for vitamin D_2_ [13].

Endogenous production in the skin is stimulated by exposure to ultraviolet B radiation, resulting in the synthesis of biologically-inactive vitamin D_3_ from 7-dehydrocholesterol. This vitamin D then binds with the vitamin D binding protein and albumin and is transported in the blood. In the liver, vitamin D is hydroxylated to 25-hydroxyvitamin D by cytochrome P450 enzymes (CYP2R1 and CYP27A1). Further modifications occur in kidneys, where 25-hydroxyvitamin D is converted to the biologically-active form, 1,25-dihydroxyvitamin D (calcitriol), by 1-α-hydroxylase (CYP27B1). Calcitriol level is regulated by a renal negative feedback loop and the CYP24A1 enzyme (24-hydroxylase), which inactivates calcitriol [13].

**Figure 1:**
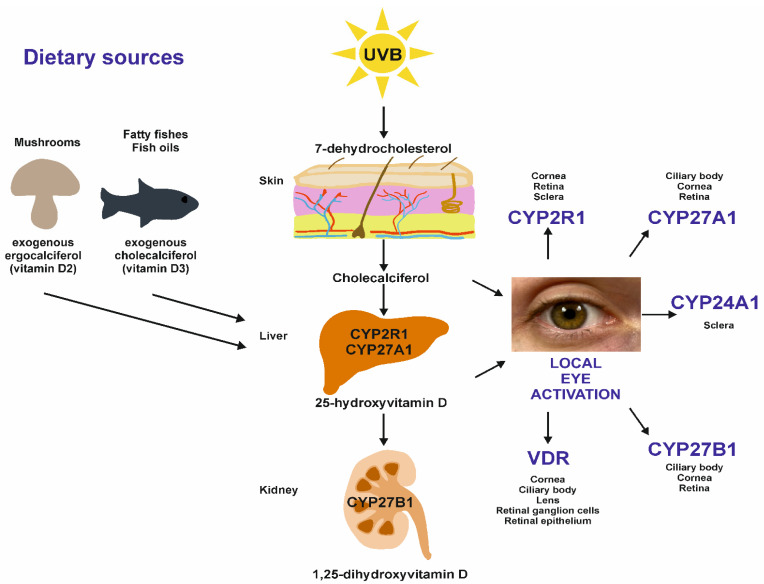
A schematic view of vitamin D metabolism, including local eye activation of vitamin D. Eye cells possess the enzymes to activate and regulate vitamin D metabolism. The figure specifies eye structures having the vitamin D receptor (VDR) and enzymes taking part in the metabolism of vitamin D. In addition, the schematic shows the dietary sources of vitamin D. Sundried mushrooms and fatty fishes, including fish oils, are the sources of ergocalciferol (vitamin D_2_) and cholecalciferol (vitamin D_3_), respectively. Both are metabolized to 25-hydroxyvitamin D_2_/D_3_ in the liver and, subsequently, to other metabolites. Nevertheless, vitamin D3 is almost twice as potent as ergocalciferol in increasing serum 25-hydroxyvitamin D, and the supplementation of vitamin D_2_ does not result in as high a blood level of 25-hydroxyvitamin D [16,17].

Vitamin D can also be metabolized locally, including in the eyes (Figure 1). Recent studies indicate that vitamin D hydroxylases are present in ocular structures such as the cornea, ciliary body, sclera and retina, suggesting that vitamin D may be an important intraocular mediator in eye diseases [14,15]. Finally, vitamin D supplementation can be used to maintain adequate concentrations.

The biological action of calcitriol, the active form of vitamin D, is mediated by VDR, a member of the nuclear hormone receptor superfamily. VDR functions as a heterodimer with the retinoid X receptor. This complex interacts with specific DNA sequences belonging to the vitamin D response element (VDRE), resulting in the activation or repression of transcription of genes influencing the function of vitamin D [18,19]. It must be emphasized that VDR is expressed in the structural elements of eye [14,20], and that certain polymorphisms of the *VDR* gene may be related to the occurrence of eye disease [21,22].

## 3. Calcitriol Analogues

As mentioned above, calcitriol takes part in the homeostasis of calcium and phosphate, and seems to be a potent agent in the treatment of many diseases. However, the therapeutic effect of exogenous calcitriol often require it to be present in supraphysiological concentrations resulting in toxic and undesirable effects, primarily hypercalcemia, hypercalciuria, excessive bone resorption and vascular calcification [23,24]. To avoid these side effects, structural analogues of calcitriol have been created, e.g., 22-oxacalcitriol and 2-methylene-19-nor-(20S)-1α,25-dihydroxyvitamin D_3_, and these have been found to have potential efficacy in ocular disorders [25,26]. These compounds deserve attention, and their detailed role in some eye diseases are described in the next section.

## 4. Vitamin D, VDR and Calcitriol Analogues in Ocular Diseases

### 4.1. Age-Related Macular Degeneration (AMD)

AMD is an acquired eye disease that affects the macula, resulting in visual impairment, and even blindness. Various risk factors have associations with development and progression of AMD, including angiogenesis, the inflammatory response, lipofuscin accumulation in retinal pigment epithelial cells, aging, smoking and sunlight exposure [27,28]. In addition, an important component of the neovascular form of AMD is the formation of new retinal blood vessels, as is apoptosis, pyroptosis and necroptosis of retinal cells, and impaired autophagy. It has been reported that vitamin D modulates programmed death pathways, and that it may influence autophagy and possess anti-angiogenic properties [29]. In addition, vitamin D deficiency is associated with a thinning of the ganglion cell complex and retinal nerve fiber layer [30]. Thus, vitamin D could modulate the course of AMD. Some studies indicate a potential association between 25-hydroxyvitamin D deficiency in serum, possibly associated with low vitamin D intake, and the risk of AMD, including the neovascular form [31,32,33,34]. In addition, some studies suggest that high levels of 25-hydroxyvitamin D can decrease the risk of AMD [35,36]. Nevertheless, most available studies, including meta-analyses, do not indicate that high serum vitamin D levels have a significant protective influence against the occurrence of any stages or subtypes of AMD [37,38,39,40,41,42,43]. In addition, in one study, vitamin D was not found to have any significant overall effect on the incidence and progression of AMD [44]. While it is reasonable to expect discrepancies between studies as a result of heterogeneity in study procedures and lack of longitudinal designs, the literature does not provide any clear evidence of a definitive association between serum 25-hydroxyvitamin D level and AMD

### 4.2. Diabetic Retinopathy (DR)

One common microvascular ocular complication of diabetes mellitus that remains the leading cause of preventable blindness is diabetic retinopathy (DR). As of 2010, this eye disorder affected over 100 million patients worldwide, and this number is expected to almost double by the year 2030 [45,46]. Studies indicate that that patients with DR had lower serum levels of vitamin D compared with those without [47] and that vitamin D (25-hydroxyvitamin D) deficiency is significantly associated with an almost twofold greater risk of DR [48,49,50,51,52,53], especially the proliferative form [54,55]. This dependence has been observed for patients with type 1 and type 2 diabetes mellitus [56,57,58]. Furthermore, it has been proposed that a serum concentration of 25-hydroxyvitamin D ≤ 18.6 ng/mL may serve as a sensitive and specific indicator for the proliferative type among subjects with DR [59]. Interestingly, endogenous vitamin D_3_ metabolites, such as 1,25-dihydroxyvitamin D, 25-hydroxyvitamin D and 24,25-dihydroxyvitamin D, could be better for predicting DR than total vitamin D level [60].

These findings regarding vitamin D deficiency in patients with DR suggest that vitamin supplementation may offer a protective effect. Indeed, vitamin D has been found to have neuroprotective properties, and its deficiency led to thinning and reduction of mean retinal nerve fiber layer thickness in early-stage DR subjects [61]. In addition, the protective effect of vitamin D in DR may also result from its ability to reduce cholesterol level, improve HDL cholesterol concentration, inhibit the activity of pro-inflammatory cytokines or pro-angiogenic and fibrotic factors, and downregulate ROS production, which play an important role in the pathogenesis of DR [62,63,64,65].

On the other hand, meta-analyses suggest that some polymorphisms of the *VDR* gene may also act as susceptibility markers for predicting the risk of DR and for earlier diagnosis of eye disease. Polymorphisms *Bsm*I, *Apa*I, and *Fok*I of the *VDR* gene are all significantly associated with DR susceptibility [66,67].

### 4.3. Optic Neuritis (ON)

ON is an inflammatory condition of the optic nerve and is a frequent cause of acute injury in adults and children. Its most common etiology is multiple sclerosis (MS) [68]. As vitamin D insufficiency appears to be a risk factor for the development of MS [69], it is possible that vitamin D may indirectly have an impact on the occurrence of ON and related changes. ON is typically associated with a thinning of the retinal nerve fiber layer (RNFL) caused by declines in axonal and macular volume [70]. Studies have found that vitamin D supplementation does not protect from the loss of RNFL and macula thickness in patients with unilateral ON (i.e., who do not fulfill the McDonald criteria for MS) concomitant with vitamin D insufficiency [70]. Nevertheless, ON may be the first manifestation of MS and occur before a full diagnosis of MS itself. Subjects with acute monosymptomatic ON are often characterized by low 25-hydroxyvitamin D levels in serum; however, these low levels of vitamin D are not correlated with the severity of ON [71]. Nevertheless, in ON patients with a low serum level of vitamin D but without a diagnosis of MS, vitamin D supplementation may delay the occurrence of secondary ON and the subsequent conversion to MS [72]. However, vitamin D levels lower than 30 ng/mL did not appear to be associated with RNFL and macular volume thickness in subjects with MS who were not affected by ON [73]. Therefore, the protective effect of vitamin D in ON is uncertain and requires further clinical trials.

### 4.4. Retinal Vein Occlusion (RVO)

One of the most common second causes of retinal vascular abnormality, and a frequent sight-threatening disorder, is retinal vein occlusion (RVO). This disease is classified as central RVO (CRVO), hemi central RVO (HCRVO) and branch RVO (BRVO) depending on the location of occlusion of the vein [74,75]. As vitamin D deficiency is believed to be associated with endothelial dysfunction and vascular diseases [76], RVO patients could have low levels of vitamin D in their serum. Indeed, significantly lower mean 25-hydroxyvitamin D serum levels have been noted in Indian subjects with RVO compared to controls; however, subjects with CRVO and BRVO did not demonstrate significant differences in mean vitamin D level compared to controls [77]. Interestingly, among CRVO patients, the mean concentration of 25-hydroxyvitamin D can be statistically significant in subjects aged under 75 years [78]; therefore, vitamin D level may be a helpful indicator for the prophylaxis, therapy or diagnosis of CRVO in this group.

### 4.5. Myopia

Myopia is the most frequent eye disorder worldwide and is a refractive anomaly. It is primarily caused by an increase in the axial length of the eyeball and elevates the risk of other ocular diseases. Typically, myopia starts in childhood [79,80]. Data indicate that the prevalence of longer axial length and myopia is significantly higher in children, young adults and adults with vitamin D deficiency compared to those with sufficient levels [81,82,83,84,85,86]. However, the relationship between myopia and vitamin D has been contradicted in some studies [87,88,89,90,91]. This discrepancy may result from other factors affecting vitamin D metabolism, such as the time spent outdoors (ultraviolet radiation exposure). It has been demonstrated that increased time spent outdoors, and ultraviolet B exposure, are effective in preventing the onset of myopia and slowing the myopic shift in refractive error associated with reduced myopia [92,93]. Further longitudinal studies are needed to determine whether higher serum 25-hydroxyvitamin D concentration is protective against myopia.

### 4.6. Retinoblastoma

Retinoblastoma is the most common intraocular cancer in children, and new options and strategies for disease therapy are being sought [94]. Initial studies indicate that retinoblastoma cells have VDR expression [95,96]. Treatment with calcitriol has been found to inhibit Y79 cell growth, inducing apoptosis by increasing the level of Bax protein and decreasing that of Bcl-2 protein [95,96]. Calcitriol was also able to induce cell cycle arrest in the G0/1 phase [96]. Moreover, 1,25-dihydroxyvitamin D significantly limited retinoblastoma growth in athymic mice with subcutaneously-injected cancer cells, as well as tumor angiogenesis in a transgenic retinoblastoma murine model [97,98]. However, calcitriol treatment was found to be toxic, resulting in elevated mortality, hypercalcemia and kidney damage [95,99]. Thus, there is a need for compounds that can act as VDR agonists but with lower calcemic effects. Such candidates include the calcitriol analogs 1,25-dihydroxy-16-ene-23-yne-vitamin D_3_, 1α-hydroxyvitamin D_2_ and 2-methylene-19-nor-(20S)-1α-hydroxybishomopregnacalciferol [95,99,100,101]. It must be emphasized that low doses of calcitriol could consolidate the effect of chemotherapy, one of the therapeutic options of retinoblastoma, and improve treatment effectiveness. In addition, the combination of cisplatin and calcitriol significantly inhibited tumor growth in athymic mice with subcutaneously injected human Y79 retinoblastoma cells without any increase in mortality and with minimal nephrotoxicity [102].

### 4.7. Uveal Melanoma (UM)

UM is a malignant and common primary intraocular tumor with a significant propensity to metastasize in adults. In almost all cases, patients with liver metastasis die within six months, and the median survival time after diagnosis of metastasis is only 3.6 months [103,104,105]. Currently, effective adjuvant therapy is not available to prevent metastases, and neither is there any successful treatment once metastases have developed [106]. Due to the lack of any efficient therapy, new strategies for UM prevention and control are urgently required. Studies show that low vitamin D status is associated with an increased risk of cancer and poor prognosis [107]. Recent studies indicate that components of the vitamin D metabolism, such as VDR, CYP27B1 and CYP24A1, are present in the UM cells [108]. Moreover, a marked reduction of the VDR expression is inversely correlated with melanin level in UM cells, as well as an aggressive UM profile, which contributes to an increased risk of metastases and poorer prognosis [108]. Interestingly, one of the most common susceptibility factors for UM is the inability to tan [109]; the ultraviolet radiation needed for the synthesis of vitamin D appears to have a protective activity against UM [110]. Furthermore, compounds such as calcitriol and calcidiol, i.e., the biologically-active metabolites of vitamin D and its precursor, have been found to sensitize melanoma cells to radiation used in the therapy of UM [111].

Clearly, there is a possible role for vitamin D and its signaling elements in the treatment of UM. Nevertheless, little is known about the impact of vitamin D on the development and progression of UM, especially considering the detailed mechanisms of its action.

### 4.8. Non-Infectious Uveitis

Uveitis is an intraocular inflammatory condition of the uvea (i.e., the iris, ciliary body, and choroid) which may lead to blindness. Uveitis can be classified depending on the primary localization of the inflammation in the eye (as anterior, intermediate and posterior uveitis) or etiology (as infectious and non-infectious). Uveitis may also be clinically divided into active and inactive forms. It is important to note that non-infectious uveitis is an immune-mediated eye disorder associated with systemic diseases such as sarcoidosis [112]. Studies have revealed an association between hypovitaminosis D and an elevated risk of non-infectious uveitis. For example, vitamin D deficiency contributes to higher risk of uveitis in juvenile idiopathic arthritis [113]. Subjects with normal levels of vitamin D had 21% lower odds of non-infectious uveitis than those with low levels of vitamin D [114]. In addition, significantly decreased blood serum levels of 25-hydroxyvitamin D were detected in patients with non-infectious anterior and non-infectious acute anterior uveitis compared to healthy controls [115,116], as well as in subjects with active non-infectious uveitis compared to inactive non-infectious uveitis patients. Vitamin D supplementation and exposure to the sun were associated with lowered disease activity in patients with vitamin D deficiency [117]. However, every unit (1 ng/mL) increase in vitamin D level was found to be associated with 4% to 5% lower odds of developing non-infectious uveitis depending on the literature source [115,118]. Interestingly, patients with sarcoidosis-related uveitis demonstrated elevated median serum levels of 1,25-dihydroxyvitamin D, compared to those with uveitis due to other causes (132.4 vs. 108.0 pmol/L). In addition, patients with sarcoidosis-induced uveitis had a higher median ratio of 1,25-dihydroxyvitamin D/25-hydroxyvitamin D (4.17 vs. 2.56): a ratio higher than 3.5 was found to be associated with the diagnosis of ocular sarcoidosis, with 68% sensitivity and 78% specificity [119].

Hence, vitamin D may play a role in the development and course of non-infectious uveitis; however, no detailed causal relationship has been identified. Further studies are necessary to determine the efficacy of vitamin D supplementation to mitigate and reduce risk of non-infectious uveitis.

### 4.9. Vogt-Koyanagi-Harada Disease (VKHD)

VKHD is a rare granulomatous autoimmune disorder that affects tissues and organs containing melanocytes: primarily the eye, inner ear, skin hair or meninges. It is characterized by panuveitis, which results in severely reduced visual acuity or even blindness if not treated appropriately. It is also accompanied by a varying degree of auditory, neurological and cutaneous manifestations. VKHD most frequently affects people from Asia, Latin America and the Middle East, as well as Native Americans [120]. It is known that vitamin D deficiency may be involved in the development of autoimmune diseases [121]. Patients with active VKHD were found to have lower serum levels of 1,25-dihydroxyvitamin D compared to subjects with inactive VKHD and healthy controls. Moreover, 1,25-dihydroxyvitamin D was found to inhibit the proliferation of peripheral blood mononuclear cells and CD4^+^ T cells, and to slow the production of interleukin (IL)-17 and interferon gamma (IFN-γ) by these cells [122].

Some polymorphisms in the genes of the vitamin D pathway may increase the occurrence of VKHD. One study did not find any polymorphisms in genes such as *VDR*, *CYP24A1* and *CYP27B1* that could contribute to VKHD; however, one polymorphism (c.852G > A; p.284 M > I) detected in *CYP2R1* appears to be associated with disease and may be pathogenic [123].

Hence, vitamin D may be involved in the development of VKHD, and one CYP2R1 polymorphism may have a causative role. Nevertheless, studies on a larger cohort of patients are necessary to confirm these observations.

### 4.10. Glaucoma

Glaucoma is a leading cause of irreversible vision loss worldwide. It is an optic neuropathy characterized by progressive degeneration of retinal ganglion cells. It is also associated with slow or difficult outflow of aqueous humor, elevated intraocular pressure (IOP) and damage of the trabecular meshwork [124,125]. Interestingly, 1α,25-dihydroxyvitamin D_3_ is able to protect against oxidative stress-induced damage to the trabecular meshwork known to occur in glaucoma, suggesting that vitamin D may have therapeutic properties [124]. In addition, the same compound has been found to reduce IOP in non-human primates [25]. Unfortunately, this result has not been confirmed in humans: no difference in IOP was found between healthy subjects with low and high serum 25-hydroxyvitamin D levels. Furthermore, vitamin D supplementation in the patients with low serum concentration of 25-hydroxyvitamin D did not change IOP [126].

Interestingly, except for a group of postmenopausal women, vitamin D deficiency may be considered an independent risk factor for open-angle glaucoma. A reverse J-shaped association was found between serum 25-hydroxyvitamin D levels and the risk of open-angle glaucoma, and patients with glaucoma, including open-angle glaucoma, were characterized by lowered serum 25-hydroxyvitamin D (by about 15% compared to the controls) [127,128,129]. Open-angle patients were also found to have significantly lower serum levels of calcitriol, another metabolite of vitamin D, compared with the age-matched controls [130]. Moreover, the serum level of vitamin D could serve as a marker of the severity of primary open-angle glaucoma, as patients with advanced glaucoma had decreased concentrations of 25-hydroxyvitamin D in comparison to early glaucoma and healthy subjects [131].

### 4.11. Cataract

Cataract is a common ophthalmic disorder that is associated with clouding of the lens [132]. Available data show that almost 100 million people worldwide are affected by cataract. The possible risk factors include increasing age, female sex, eyeball trauma, ultraviolet-B-exposure, cigarette smoking, diet with a high glycemic index, malnutrition, and genetic factors [133]. Other diseases such as diabetes mellitus, renal impairment, metabolic syndrome and arterial hypertension increase the risk of cataract [133]. In addition, vitamin D may have an effect on cataract. Patients with age-related cataract dad lower mean serum levels of 25-hydroxyvitamin D compared to controls (7.6 ± 5.5 ng/mL vs. 18.5 ± 9.6 ng/mL), and this change was statistically significant. In addition, among different types of age-related cataract, cases of nuclear cataract were characterized by the lowest level of vitamin D [134]. Moreover, vitamin D deficiency in serum was found to be associated with age-related cataract, including the early form of disease [135,136]. Interestingly, studies indicate that the risk of nuclear cataract is inversely correlated with the serum level of 25-hydroxyvitamin D, with it appearing to have a protective role against nuclear cataract [137,138].

Although posterior subcapsular cataract resembles hypocalcemic cataract, the relationship between low serum vitamin D levels and posterior subcapsular cataract formation remains unclear. Brown et al. (2015) suggest that vitamin D deficiency may contribute to the development of posterior subcapsular cataract, and that some comorbidities and non-ophthalmic interventions are associated with posterior subcapsular cataract in the presence of decreased levels of 25-hydroxyvitamin D. Interestingly, some patients with early-stage posterior subcapsular cataract co-existing with vitamin D deficiency had a resolution of changes in the lens following daily supplementation of 5000 IU of 25-hydroxyvitamin D over a 2-year follow-up period [139]. In contrast, no vitamin D deficiency was later observed in patients with posterior subcapsular cataract [140]. In turn, diabetic cataract was characterized by significantly higher levels of 25-hydroxyvitamin D in aqueous humor compared to senile cataract [141].

Vitamin D may well play a role in lens metabolism. However, further extensive trials are needed to explain the correlation between vitamin D concentration and cataract, and to understand the detailed mechanisms of vitamin D activity in this regard.

### 4.12. Scleritis

Scleritis is an infrequent inflammatory disorder of the sclera that may be caused by infectious factors, trauma, drugs or irradiation, and one that frequently accompanies immune-mediated diseases. The immune system appears to play an important role in the pathogenesis of non-infectious scleritis [142]. Therefore, there is an association between scleritis and vitamin D, which is able to suppress the immune response mainly by the modulation of T lymphocyte activity. Multivariate analyses revealed a statistically significant link between non-infectious scleritis and decreased 25-hydroxyviatmin D levels at any time point before or after the onset of scleritis (28.1 ng/mL vs. 34.4 ng/mL in controls, *p* = 0.009) and any time point before (24.1 ng/mL vs. 34.4 ng/mL in controls, *p* = 0.033). In addition, the same analysis showed that the odds of developing scleritis were 4% lower for every unit (1 ng/mL) increase in the vitamin D level (OR = 0.96, 95% CI = 0.93–0.99, *p* = 0.009) [118]. Further studies are necessary to establish the potential role of vitamin D supplementation in the development of scleritis.

### 4.13. Dry Eye Syndrome (DES)

DES is a common ophthalmic disorder affecting the ocular surface, which is marked by persistent symptoms. One of the main mechanisms contributing to the development of disease is the inflammatory reaction [143]. Studies indicate that calcitriol, an active metabolite of vitamin D, is able to inhibit dry eye-related corneal inflammation and apoptosis in in vitro and in vivo experimental models of DES, highlighting the protective role of vitamin D [144,145,146]. DES patients had significantly lower serum levels of 25-hydroxyvitamin D, and this correlated with ocular surface disease index [147,148,149,150]. Furthermore, DES patients also displayed significantly lower concentrations of vitamin D in their tears [151], which may result from the fact that the vitamin D deficiency is associated with tear hyperosmolarity, a dysfunction of tear film and reduction of Schirmer’s test value [152,153]. Serum level of 25-hydroxyvitamin D was also found to correlate positively with tear break-up time and tear secretion [154].

In contrast, some studies did not show any association between DES and lowered vitamin D levels [155,156]. Vitamin D (cholecalciferol) supplementation was found to improve tear break-up time, tear secretion, eyelid margin hyperemia and the severity of symptoms in patients with DES refractory to conventional treatment concomitant with vitamin D deficiency [157]. In addition, cholecalciferol supplementation enhanced the efficacy of artificial tears and hyaluronate, which are used in the therapy of DES, and may be a useful adjuvant treatment for subjects with DES refractory to topical lubricants [158]. Furthermore, two single nucleotide polymorphisms (Foklrs2228570 and Apal-rs7975232) in the VDR gene seem to be associated with DES and may potentially be used in the diagnosis of disease [22].

### 4.14. Vernal Keratoconjunctivitis (VKC)

VKC is a chronic, allergic, inflammatory disease of the tarsal and/or bulbar conjunctiva, which occurs seasonally and mainly in the pediatric population [159]. Data show that vitamin D may mitigate allergic diseases [160]. It has been demonstrated that children with VKC have lower serum levels of 25-hydroxyvitamin D in comparison to healthy controls. In addition, a significant correlation has been confirmed between vitamin D level and VKC severity, including VKC objective score and basophils in conjunctival scraping [161]. In contrast, a more recent study confirmed an inverse correlation between serum vitamin D levels and VKC severity; however this difference was not statistically significant [162]. Another study found decreased mean serum concentrations of 25-hydroxyvitamin D in children affected by VKC. In addition, the VKC-children tended to spend less time outdoors during daylight compared to controls (160.7 ± 65.9 vs. 229.5 ± 101.2 min), suggesting that a reduced level of vitamin D may play a role. In addition, a statistically significant correlation was observed between serum 25-hydroxyvitamin D levels and time spent outdoors [163].

Severe VKC is often treated using immunomodulatory drugs, such as cyclosporine or tacrolimus [164]. Furthermore, studies indicate that treatment with 1% cyclosporine or 0.1% tacrolimus as eye drops may contribute to the improvement of VKC signs and symptoms, and that these changes are associated with an increase of 25-hydroxyvitamin D level in serum. This rise was higher in the children with limbal VKC than the tarsal form and could result from the fact that limbal VKC is less symptomatic [161,165]. The above data require confirmation and verification through large-scale clinical trials; however, their findings may provide a greater insight into the pathogenesis of VKC and creation of customized therapy.

### 4.15. Keratoconus

Keratoconus is a common, progressive, ectatic, degenerative disease of the cornea. It manifests as a thin cone resulting in refractive errors, such as irregular astigmatism or myopia, with impairment of visual acuity. Although the detailed pathogenesis of keratoconus formation remains unclear, environmental and genetic factors are believed to have an important role, and proposed mechanisms include inflammatory reaction, oxidative stress and proteolytic degradation in the corneal stroma [166,167].

Recent data suggest that keratoconus may be also associated with disturbances of the immune system, and the systemic inflammatory response related to autoimmune diseases can induce the onset of eye disease [168]. As vitamin D is known to have immunomodulating properties [169], some studies have attempted to identify a relationship between its level and the onset of keratoconus. One study found that patients with keratoconus had significantly decreased serum levels of vitamin D compared to age- and sex-matched controls; however, no statistically significant correlation was found between 25-hydroxyvitamin D concentrations and keratoconus severity, based on Pentacam measurements including anterior curvature [170]. Similarly, Zarei-Ghanavati et al. (2020) reported that although severe keratoconus (Krumeich criteria stage IV) was associated with the lowest level of 25-hydroxyvitamin D, no significant differences were observed among individual groups of patients [171].

Reduced vitamin D levels have been found to significantly increase the probability of non-progressive and progressive keratoconus compared to controls by 1.23 and 1.29 times, respectively. Nevertheless, despite both groups demonstrating significantly lower concentrations of vitamin D in comparison to controls, no significant differences were found between non-progressive and progressive disease [172].

There is clearly a need for further studies investigating the potential relationship between keratoconus and vitamin D and to determine whether vitamin D supplementation may prevent or inhibit the course of disease.

### 4.16. Pterygium

Pterygium is a chronic disease of the anterior segment of the eye characterized by benign, uncontrolled, fibrovascular growth of the bulbar conjunctiva across the cornea, leading to impairment of visual acuity [173,174]. Its pathophysiology is not fully known. Inflammation and angiogenesis are considered important in the course of disease. However, chronic exposure to ultraviolet light is believed to have a significant causative relationship with pterygium. Moreover, a strong link has been evidenced between disease and geographical latitude: the prevalence of pterygium is inversely associated with latitude, indicating that ultraviolet radiation in the development of this eye disorders. This may also indirectly suggest that vitamin D plays a role in the formation of pterygium [175,176,177,178].

This was confirmed by Jee et al. (2016), who found that patients with pterygium had higher serum levels of 25-hydorxyvitamin D even after controlling for sunlight exposure time, and these changes were statistically significant. This positive association was found for both sexes. Nevertheless, it must be emphasized that despite significant difference between the patients with and without pterygium, those with eye disease had vitamin D insufficiency, with a mean serum 25-hydroxyvitamin D level of 20.4 ng/mL [179].

These results were later confirmed by another group, who showed significantly higher serum levels of 25-hydroxyvitamin D to be present in men with pterygium compared to healthy male controls. Importantly, the men with pterygium with more outdoor activity had more elevated concentrations than those with dominant indoor activity. No such relationship was observed in controls, indicating that vitamin D has a potential role in the development of pterygium. However, no such differences were found between female patients and healthy subjects [180]. Another study examined the relationship between pterygium, sun exposure, and serum 25-hydroxyvitamin D level in South Korean adults. The frequency of pterygium was found to be increased in elderly subjects and those who lived at low geographical latitudes. In addition, these patients had higher serum levels of 25-hydroxyvitamin D, suggesting that this may be positively correlated with the prevalence of pterygium [178]. However, a recent study in 2021 did not identify any changes in vitamin D concentration in both serum and tear fluid between the subjects with pterygium and healthy subjects [181]. None of the trials mentioned above evaluated or described the mechanisms underlying the relationship between vitamin D and pterygium. Although vitamin D has pro-health properties, including anti-inflammatory activities, it appears to have an insufficient impact on pterygium to prevent disease. Furthermore, many studies have not compared vitamin D concentration with characteristics of pterygium, such as length or histopathological evaluation. Therefore, further clinical trials are warranted.

Interestingly, a recent study did not find any difference between patients with pterygium and healthy subjects with regard to serum vitamin D level; however, VDR protein expression was found to be elevated in endothelial cells of micro-vessels, subepithelial stromal and intravascular inflammatory cells associated with pterygium compared to adjacent healthy conjunctival tissue [182]. Immunohistochemical assays found VDR localization to differ significantly in the pterygium cells compared to normal conjunctival cells. In healthy conjunctiva, VDR was localized mainly in the cytoplasm, while in pterygium cells, VDR was co-localized in the nucleus and cytoplasm. Hence, the nuclear signaling pathways related to VDR may be engaged in the pathogenesis of pterygium [183]. Further analyses and clinical trials are needed to standardize the role of VDR in pathogenesis and the development of pterygium.

### 4.17. Thyroid Eye Disease (TED)

Graves’ disease, the most common cause of thyrotoxicosis, is an autoimmune disease affecting the thyroid gland. It is characterized by the presence of autoantibodies directed against antigens in the thyroid. These may cross-react with orbital antigens leading to TED, known also as Graves orbitopathy or thyroid-related orbitopathy. It is an inflammatory-fibrotic orbitopathy that includes orbital tissues, mainly extraocular muscles and orbital fat, causing diplopia, photophobia, exposure keratopathy and eye pain; if left untreated, the condition can generate compressive optic neuropathy [184,185].

Studies indicate that the vitamin D insufficiency may contribute to disturbances in immune system activity, which is an important factor in the development of autoimmune diseases [121]. Meta-analyses indicates that low vitamin D levels are related to the occurrence of Graves’ disease, a lower likelihood of remission and a higher recurrence rate [186,187]. Decreased serum concentrations of 25-hydroxyvitamin D have also been noticed in patients with TED. A retrospective study by Sadaka et al. (2019) found that 20% of TED-patients had vitamin D deficiency (a level below 20 ng/mL), and 31% had insufficiency (a level between 20 and 29 ng/mL). However, these results were obtained using a relatively small sample size, and no correlation was made with clinical disease activity or with an unaffected cohort [188].

Assessment of vitamin D level and its supplementation may play an important role in the early management of Graves’ disease by preventing the development of TED. Patients with Graves’ disease concomitant with TED have lower serum levels of 25-hydroxyvitamin D compared to those with Graves’ disease but without TED [189]. Research efforts are now directed toward identifying characteristics, including gene polymorphisms, which could modify the risk of TED and be associated with its occurrence; these could be used to facilitate early diagnosis. Maciejewski et al. (2020) found a C gene polymorphism rs2228570 (*Fok*I) in *VDR* to occur more frequently in patients compared to unaffected subjects; this may be a risk factor contributing to the development of TED in patients of Caucasian origin with Graves’ disease [190].

### 4.18. Benign Essential Blepharospasm (BEB)

BEB is a cranial dystonia characterized by hyperactivity and sustained, involuntary spasms of muscles around the eyes, such as orbicularis oculi, corrugator and procerus. This ocular disorder affects approximately 1.4–13.3 cases per 100,000 people, mainly women, and the typical onset of the disease occurs between the fifth and seventh decades of life. Although the detailed causes of BEB remain unknown [191,192,193], they may be associated with disturbances in the regulation of intracellular and extracellular ionized calcium (Ca^2+^), an ion responsible for triggering muscle contraction [194]. Interestingly, patients with BEB demonstrate significantly lower serum calcium levels than healthy subjects [194]. This may indicate decreased vitamin D concentrations, whose deficiency is related to lowered ionized calcium levels and even hypocalcemia [195]. In one study, no significant differences in serum 25-hydroxyvitamin D level were found between BEB-patients and unaffected subjects, and only a moderate negative correlation was found between vitamin D levels and the severity of BEB based on the Jankovic score [194]. However, in another study, BEB patients demonstrated significantly lower 25-hydroxyvitamin D and calcium levels, which may suggest a potential cause of this disorder [196]. Further long-term prospective clinical trials are necessary to determine the role of vitamin D in the involvement of BEB pathophysiology.

## 5. Potential Mechanisms of the Vitamin D Action against Ocular Diseases

The ocular diseases described above have various risk factors, etiology and ethiopathogenesis. Inflammation, oxidative stress, angiogenesis, and apoptosis play an important role in the development of most of them [94,143,174]. It was shown that vitamin D was able to mitigate the inflammatory response mainly by inhibition of nuclear factor kappa B (NF-κB) signaling pathway, modulation of the immune cells activity and suppression of pro-inflammatory factor expression, such as cyclooxygenase-2 among others, resulting in a reduction of the prostaglandins level. In addition, vitamin D has been found to modulate apoptosis and downregulate the expression of vascular endothelial growth factor (VEGF) inhibiting angiogenesis [197]. Pro-health effects have also been confirmed for eye disorders. Vitamin D counteracted oxidative stress induced by hydrogen peroxide (H_2_O_2_) in human retinal pigment cells, and mitigated the inflammation induced by H_2_O_2_ through a decrease of the protein expression of interleukin (IL) 1β (IL-1β), IL-8, tumor necrosis factor alpha (TNF-α). Furthermore, the anti-inflammatory effect of vitamin D was confirmed in another in vitro model using lipopolysaccharide (LPS)-stimulated human retinal pigment epithelial cells. These data show that vitamin D may inhibit retinal diseases, such as AMD and DR, limiting inflammation [198,199]. The intraperitoneal administration of calcitriol and 22-oxacalcitriol significantly attenuated lesion volume in laser-induced choroidal neovascularization using a mouse model. Therefore, vitamin D and calcitriol analogues may have potential as an interventional treatment for ophthalmic neovascular indications [26]. In hyperosmotic stress-induced human corneal epithelial cells, calcitriol inhibited the reactive oxygen species (ROS)-NLR family pyrin domain containing 3 (NLRP3)-IL-1β signaling axis via activation of the Nrf2-antioxidant pathway, indicating that it may prevent and mitigate DES-related corneal inflammation and oxidative stress at an early stage [144]. Calcitriol is also able to prevent human corneal epithelial cells from apoptosis via activation of autophagy via the VDR pathway [146]. The potential therapeutic mechanism of calcitriol was also revealed in glaucoma. This compound attenuated oxidative stress-induced damage in human trabecular meshwork cells by inhibiting the transforming growth factor beta (TGF-β)-SMAD family member 3 (SMAD3)-VDR pathway [124]. On the other hand, VDR, including its activation and inhibition, may play an important role in the development and progression of ocular diseases. For example, inhibition of VDR exerted a protective role in high-level glucose-induced damage of retinal ganglion cells by activating the signal transducer and activator of the transcription 3 (STAT3) pathway, indicating the potential role of VDR in DR [200]. Mechanisms of vitamin D action in ocular diseases are complex and not fully known. It seems that cholecalciferol and calcitriol may exert anti-inflammatory, anti-oxidative and anti-angiogenic effects accompanied by a reduction of development and progression of ocular diseases. Nevertheless, further studies are necessary to explain the detailed mechanisms of vitamin D action in eye disorders and their association with the VDR activity.

## 6. Summary and Conclusions

Based on the papers included in this review, it appears that vitamin D level may be associated with a range of eye diseases, including DR, ON, RVO, myopia, UM, non-infectious uveitis, VKHD, glaucoma, cataract, scleritis, DES, VKC, keratoconus, pterygium, TED and BEB. Measurement of vitamin D level, primarily 25-hydroxyvitamin D, could be a practical marker of the clinical course and severity of some ocular disorders, or could even be used to predict their risk. The data described in this review indicate that vitamin D has some pro-health properties which can be used against the ocular diseases. Therefore, vitamin D may be an agent supporting the available treatment of eye disorders. For example, it has been suggested that deficiency of the vitamin D occurs in patients with Sjögren’s Syndrome-related dry eye, and this may result in a modulation of the clinical course [201]. That is why vitamin D may be considered as a modulator of the clinical course of ocular diseases. In addition, vitamin D may serve as marker of the advancement of the disease, since its level is correlated with severity of the symptoms [148]. Thus, vitamin D may be a tool in the diagnostic and therapeutic process, although further studies are required to confirm this. In addition, some *VDR* gene polymorphisms may serve as prognostic markers. Interestingly, calcitriol analogues appear to limit the development and progression of retinoblastoma.

The studies a described above have some limitations. First, vitamin D deficiency is becoming an increasing problem worldwide, and was observed in most study participants, even those without apparent disease. It is also not always clear whether vitamin D deficiency is the cause or consequence of a disorder, and the molecular mechanisms of vitamin D action remain poorly understood. Other significant limitations of the studies include the small number of patients, high heterogeneity of selected groups and variation in previous vitamin D supplementation. In addition, considerable variation has occurred in the methods used to measure vitamin D serum concentration, environmental conditions and individual factors (including sunlight exposure, physical activity). Most importantly, vitamin D levels vary seasonally, and this would affect the results depending on when the study was performed. Therefore, further randomized controlled trials are needed to clarify conflicting results.

Selected clinical studies and trials investigating the link between vitamin D, VDR and ocular diseases are summarized in Table 1.

## Figures and Tables

**Table 1:** Summary of clinical studies and trials investigating the relation between vitamin D and eye diseases.

Type of Study/ClinicalTrials.gov Identifier/Phase (If Specified)	Participants/Enrollment/DeMographics of Population	Method	Findings	Ref
Age-related macular degeneration				
Case-control study	161 neovascular AMD cases from two university hospitals and 369 population-based control subjects from a cohort study	Brief-type self-administered questionnaire on diet history, which required respondent recall of the usual intake of 58 foods during the preceding month	Logistic regression analysis showed that low intake of vitamin D was associated with neovascular AMD (Trend *p* < 0.002)	[32]
Clinical case–control pilot study	96 Korean patients: 30 with late AMD, 32 with early AMD, and 34 normal controls	Measurement and comparison serum 25(OH)D levels	Serum vitamin D deficiency may elevate the risk of early (OR = 3.59; 95%CI 0.95–13.58; *p* = 0.06) and late AMD (OR = 3.61; 95%CI 1.04–12.51; *p* = 0.043) and may also be associated with subretinal fibrosis	[33]
Cross-sectional study	95 patients with exudative AMD and 95 healthy age- and sex-matched controls	Measurement and comparison serum 25(OH)D levels	Significant lower 25(OH)D levels in patients with AMD compared to the control subjects (*p* = 0.042)	[34]
Population-based, cross-sectional study	17,045 Korean subjects older than 40 years	Standardized interviews, evaluation of blood 25-(OH)D levels and comprehensive ophthalmic examinations	Inverse association between high level of blood 25(OH)D with late AMD in men but not women	[35]
Population-based, prospective analysis	1225 (196 African American; 1029 Caucasian)	Evaluation of blood 25-(OH)D levels and ophthalmic examinations	Logistic regression showed that high 25(OH)D concentrations (>70 nM) may be associated with reduced odds of incident early AMD	[36]
Cross-sectional study	1045 members with AMD and 8124 without AMD	Comparison of serum 25(OH)D levels between the two groups	No association was detected between 25(OH)D levels and the presence of AMD	[37]
Population-based study	697 (264 men, 433 women)	Assessment of associations between AMD and plasma 25(OH)D status using generalized estimating equation logistic regressions	Lack of specific role of vitamin D in AMD	[38]
Meta-analysis	Literature database	Identification of the association between serum vitamin D levels and AMD risk	Lack of evidences to inverse association between serum vitamin D levels and any stages and subtypes of AMD risk	[39]
Population-based, cross-sectional study	2137 without AMD, 2209 with early AMD, 150 with late AMD, of whom 104 with neovascular AMD	Comparison of serum 25(OH)D levels	No linear association between 25(OH)D and early or late AMD or neovascular AMD	[40]
Cross-sectional study	9734 (7779 Caucasians, 1955 African American	Secondary data analysis of already existing data from the AtherosclerosisRisk in Communities Study	Lack of association between vitamin D status and early AMD	[41]
Pilot study	Treatment group with 15 subjects and control, group with 15 subjects	Measurement and comparison serum 25(OH)D levels	Lack of association between vitamin D status and AMD	[42]
Systematic review and meta-analysis	Literature database	Identification of the association between serum vitamin D levels and AMD risk	Lack of a definitive association between serum 25(OH)D and AMD risk	[43]
Nationwide, double-blind, placebo controlled randomized clinical trial	25,871	Supplementation of 2000 IU/day for a median 5.3 years	Lack of vitamin D supplementation on AMD incidence and progression	[45]
Diabetic retinopathy				
Cross-sectional study	30 subjects with DR and 30 subjects without DR	Measurement and comparison serum 25(OH)D levels	Lower levels of serum 25(OH)D in DR	[47]
Cross-sectional study	1790	Measurement and comparison serum 25(OH)D levels	Association between DR and prevalence of vitamin D deficiency	[48]
Population-based cross-sectional study	2113 participants aged ≤ 40 years	Evaluation of blood 25-(OH)D levels and ophthalmic examinations	Inverse association of blood 25(OH)D levels with any DR and proliferative DR only in men	[49]
Meta-analysis	Literature database	Identification of the association between serum vitamin D levels and DR	Statistically significant association between vitamin D deficiency and DR	[50]
Retrospective study	3054 Asian Indians with type 2 diabetes mellitus	Evaluation of blood 25-(OH)D levels and ophthalmic examinations	Association between lower serum 25(OH)D and increased severity of DR. Association between vitamin D deficiency and two-fold increased risk for proliferative DR	[51]
Cross-sectional study	638 patients with type 2 diabetes mellitus	Evaluation of blood 25-(OH)D levels and clinical examinations	Vitamin D deficiency is considered as a risk factor for DR	[52]
Cross-sectional study	4767 diabetic patients	Evaluation of blood 25-(OH)D levels and ophthalmic examinations	Association between lower serum 25(OH)D and higher prevalence of DR in middle-aged and elderly diabetic adults	[53]
Clinic-based, cross-sectional study	221subjects	Evaluation of blood 25-(OH)D levels and ophthalmic examinations	Diabetic subjects, especially those with PDR, have lower 25(OH)D levels than those without diabetes	[54]
Hospital-based cross-sectional study	889 type 2 diabetic patients with or without DR	Evaluation of blood 25-(OH)D levels and clinical examinations	Vitamin D deficiency is significantly associated with risk of proliferative DR	[55]
Cross-sectional study	517 subjects aged 8–20 years with type 1 diabetes mellitus	Evaluation of blood 25-(OH)D levels and ophthalmic examinations	Association between the vitamin D deficiency and increased prevalence of DR in young people with type 1 diabetes mellitus	[56]
Meta-analysis	Literature database	Identification of the association between serum vitamin D levels and DR	Association between the vitamin D deficiency and increased risk of DR patients with type 2 diabetes mellitus	[57]
Retrospective study	182 with type 1 diabetes mellitus	Evaluation of blood 25-(OH)D levels and ophthalmic examinations	Association between the vitamin D deficiency and increased prevalence of DR in patients with type 1 diabetes mellitus	[58]
Cross-sectional study	460 patients with type 2 diabetes mellitus (median age 55.2 years; age range, 30–90 years; 227 male and 233 female) and 290 non-diabetic control subjects (median age 46.1 years; age range, 30–85 years; 151 male and 139 female)	Evaluation of blood 25-(OH)D, 1,25(OH)_2_D, 24,25(OH)_2_D levels and ophthalmic examinations	Association between vitamin D_3_ metabolites and DR, whereas lack of these dependence for total vitamin D levels	[60]
Tertiary care center based cross-sectional study	Diabetes mellitus without DR (24), non-proliferative DR (24), proliferative DR (24) and controls (24)	Evaluation of blood 25-(OH)D levels and ophthalmic examinations	Serum vitamin D levels of ≤ 18.6 ng/mL is marker for proliferative DR	[59]
Systematic review and meta-analysis	Literature database	Identification of the association between *VDR* gene polymorphisms and DR susceptibility	The *VDR FokI* gene variant is associated with DR	[66]
Meta-analysis	Literature database	Identification of the association between *VDR* gene polymorphisms and DR susceptibility	The *VDR BsmI*, *ApaI* and *FokI* gene variants are associated with DR susceptibility	[4]
Optic neuritis				
Placebo-controlled randomized clinical trial	52 patients with confirmed unilateral ON aged 15–38 years and low serum 25(OH)D levels	Supplementation of 50,000 IU/week vitamin D_3_ or placebo for 6 months	Adding vitamin D to routine disease therapy had no significant effect on the thickness of RNFL or macula in patients with ON	[70]
Cross-sectional study	164 patients with monosymptomatic ON and 948 patients with MS	Evaluation of blood 25-(OH)D levels and clinical examinations	Lack of correlation between levels of vitamin D and ON severity	[71]
Doubleblind, randomized, placebo-controlled pilot clinical trial	30 patients with ON	Supplementation of 50,000 IU/week vitamin D_3_ or placebo for 12 months	Administration of vitamin D_3_ by patients with ON and low serum 25-(OH)D levels may delay the onset of a second clinical attack and the subsequent conversion to MS	[72]
Cross-sectional study	74 patients with MS	Evaluation of blood 25-(OH)D levels and ophthalmic examinations	Lack of association between vitamin D deficiency and thinning of RNFL or macular volume in MS eyes unaffected by ON	[73]
Retinal vein occlusion				
Pilot study	40 patients with RVO and 40 control subjects	Evaluation of blood 25-(OH)D levels and ophthalmic examinations	Significant lower levels of serum 25(OH)D in RVO as compared to age matched controls	[77]
Case control study (NCT01793181)	79 patents with CRVO and 144 control subjects	Evaluation of blood 25-(OH)D levels and clinical examinations	Patients under 75 years with CRVO had significantly lower 25(OH)D levels compared to the control	[78]
Myopia				
Study based on Korea National Health and Nutrition Examination Survey	2038 Korean adolescent aged 13 to 18 years	Evaluation of blood 25-(OH)D levels and ophthalmic examinations	Decreased serum 25(OH)D level was associated with myopia prevalence in Korean adolescents	[81]
Study based on the Western Australian Pregnancy Cohort (Raine) Study	221 patients with myopia, 725 nonmyopic subjects	Evaluation of blood 25-(OH)D levels and ophthalmic examinations	Decreased serum 25(OH)D level was associated with myopia	[82]
Study based on Korea National Health and Nutrition Examination Survey	15,126 Korean aged 20 years or older	Evaluation of blood 25-(OH)D levels, daily sun exposure time and ophthalmic examinations	Low serum 25(OH)D levels and shorter daily sun exposure time is independently associated with a high prevalence of myopia	[83]
Population-based prospective cohort study	2666 children aged 6 years	Evaluation of blood 25-(OH)D levels, daily sun exposure time and ophthalmic examinations	Low serum 25(OH)D is associated with axial length and risk of myopia in young children	[84]
Systematic review and meta-analysis	Literature database	Identification of the association between serum vitamin D levels and myopia	Lower 25(OH)D is associated withincreased risk of myopia	[85]
Study based on Korea National Health and Nutrition Examination Survey	25,199 subjects aged ≥ 20 years	Evaluation of blood 25-(OH)D levels and ophthalmic examinations	Serum 25(OH)D level was inversely associated with myopia in adults	[86]
Study based on the Avon Longitudinal Study of Parents and Children (ALSPAC) population-based birth cohort	Children participating in ALSPAC	Evaluation of total vitamin D, vitamin D_3_, vitamin D_2_ levels, time outdoors and ophthalmic examinations	Vitamin D may serve as biomarker for time spent outdoors without association with myopia	[87]
Mendelian randomization study based on meta-analysis of refractive error genome-wide association study	37,382 and 8376 adult participants of European and Asian ancestry, respectively	Identification of the association between serum vitamin D levels and myopia	Contribution of vitamin D levels to degree of myopia is very small and indistinguishable from zero	[88]
Study based on the Busselton Healthy Ageing Study	Community-based cohort of adults aged 46 to 69 years	Evaluation of blood 25-(OH)D levels and ophthalmic examinations	There was no substantial association between 25(OH)D levels and spherical equivalent or odds of myopia	[89]
Prospective, cross-sectional study	Children born prematurely between January 2010 and December 2011 when they reached school age between April 2017 and June 2018	Evaluation of blood 25-(OH)D levels and ophthalmic examinations	More time spent outdoors is associated with lower odds of myopia. The serum 25(OH)D concentration is not associated with myopia	[90]
Case-control study	457 myopic male cases and 1280 emmetropic male controls	Evaluation of blood 25-(OH)D levels and ophthalmic examinations	The myopia is not related to neonatal vitamin D status	[91]
Cross-sectional, population-based random study	4166 participants 65 years and older	Evaluation of vitamin D_3_ levels, time outdoors and ophthalmic examinations	Increased UVB exposure is associated with reduced myopia	[92]
Systematic review and meta-analysis	Literature database	Identification of the association between time spent outdoor and myopia	Increased time outdoors is effective in preventing the onset of myopia as well as in slowing the myopic shift in refractive error. Outdoor time is not effective in slowing progression in eyes that were already myopic.	[93]
Non-infectious uveitis				
Prospective, multicenter, observational cohort study	360 Patients ≤ 16 years of age with recently diagnosed JIA (< 12 months)	Evaluation of blood 25-(OH)D levels and clinical examinations	Vitamin D deficiency is associated with the risk for uveitis in juvenile idiopathic arthritis	[113]
Case-control study	558 patients with non-infectious uveitis and 2790 control subjects	Evaluation of blood vitamin D levels and clinical examinations	Association between hypovitaminosis D and non-infectious uveitis	[114]
Case-control study	100 patients with non-infectious anterior uveitis and 100 control subjects	Evaluation of blood 25-(OH)D levels and ophthalmic examinations	Lower vitamin D levels are associated with an increased risk of non-infectious anterior uveitis	[115]
Observational case–control study	20 patients with acute anterior uveitis and 100 consecutive, age and sex matched healthy subjects without any ocular or systemic diseases	Evaluation of blood 25-(OH)D levels and ophthalmic examinations	The patients with acute anterior uveitis have decreased 25(OH)D level	[116]
Prospective case-control study	74 patients with active uveitis and 77 patients with inactive uveitis	Evaluation of blood 25-(OH)D levels and ophthalmic examinations	Active uveitis is associated with significantly lower serum 25(OH)D levels than inactive uveitis	[117]
Retrospective case-control study	333 patients with uveitis and 329 control subjects	Evaluation of blood 25-(OH)D levels and ophthalmic examinations	Hypovitaminosis D is associated with increased risk of non-infectious uveitis	[118]
Monocentric retrospective cohort study	59 patients with uveitis	Evaluation of blood 25-(OH)D, 1,25,(OH)D levels and ophthalmic examinations	High 1,25(OH)_2_D/25(OH)D ratio is useful for the diagnosis of sarcoidosis-related uveitis	[119]
Vogt-Koyanagi-Harada disease				
Case-control study	25 patients with VKHD and 16 health subjects	Evaluation of blood 1,25(OH)_2_D levels and clinical examinations	Reduced expression of 1,25(OH)_2_D may be involved in the development of VKHD	[122]
Case-control study	39 patients with VKHD and 50 control subjects	Sequencing analysis of the *VDR*, *CYP24A1*, *CYP27B1* and *CYP2R1* genes (involved in the metabolism of vitamin D)	Detection of potentially pathogenic sequence variant in CYP2R1 may cause VKH in a subset of patients	[123]
Glaucoma				
Case-control and an intervention study	39 received vitamin D supplements, 39 received placebo and 42 control subjects	Evaluation of blood 25-(OH)D levels and ophthalmic examinations; Supplementation of 40000 IU/week vitamin D3 or placebo for 6 months for participants with low vitamin D level	No difference between IOP and low/high serum 25(OH)D levels. No significant difference between experimental and control groups	[126]
Cross-sectional study	6094	Evaluation of blood 25-(OH)D levels and ophthalmic examinations	Vitamin D deficiency Seems to be independent risk factor for open-angle glaucoma	[127]
Case-control study	150 patients with glaucoma and 164 health subjects	Evaluation of blood 25-(OH)D levels and ophthalmic examinations	Serum vitamin D status is associated with the presence but not the severity of primary open angle glaucoma	[128]
Cross-sectional and observational study	20 patients with glaucoma and 20 control subjects	Evaluation of blood 25-(OH)D levels and ophthalmic examinations	Serum Vitamin D level is statistically significantly lower in glaucoma	[129]
Case-control study	73 with glaucoma and 71 age-matched control subjects	Evaluation of blood calcitriol levels and ophthalmic examinations	Decreased calcitriol levels in glaucoma	[130]
Case-control study	357 patients with glaucoma and 178 control subjects of African descent	Evaluation of blood 25-(OH)D levels and ophthalmic examinations	Association between the level of 25-(OH)D and glaucoma severity	[131]
Cataract				
Case-control study	325 patients with cataract and 385 control subjects	Evaluation of blood 25(OH)D levels and ophthalmic examinations	Association of Serum 25(OH)D deficiency and age-related cataract	[134]
Observational cross-sectional study based on Korea National Health and Nutrition Examination Survey	18804	Evaluation of blood 25(OH)D levels and ophthalmic examinations	The age-related cataract risk is decreased in men with higher serum 25(OH)D levels	[135]
Case control study	37 patients with cataract and 53 health subjects under the age of 60 years	Evaluation of blood 25(OH)D levels and ophthalmic examinations	Vitamin D deficiency is associated with early age-related cataract	[136]
Study based on the Carotenoid Age-Related Eye Study	1278	Evaluation of blood 25(OH)D levels and ophthalmic examinations	Inverse association between serum 25(OH)D and nuclear cataract in women younger than 70 years	[137]
Study based on Korea National Health and Nutrition Examination Survey	16,086 (7093 males and 8993 females) adults aged 40 years or older	Evaluation of blood 25(OH)D levels and ophthalmic examinations	Serum 25(OH)D levels are inversely associated with the risk of nuclear cataract	[138]
Retrospective chart review study	195	Evaluation of blood 25(OH)D levels and ophthalmic examinations	Vitamin D deficiency is associated with posterior subcapsular cataract	[139]
Prospective hospital-based cross-sectional study	79 patients with posterior subcapsular cataract or age-related cataract without posterior subcapsular cataract component	Evaluation of blood vitamin D levels and ophthalmic examinations	Posterior subcapsular cataract is not associated with vitamin D insufficiency	[140]
Prospective study	87 patients with senile cataract and 49 patients with diabetic cataract	Evaluation of blood and aqueous humor 25(OH)D levels and ophthalmic examinations	Higher 25(OH)D level in aqueous humor is associated with diabetic cataract	[141]
Scleritis				
Retrospective case-control study	103 patients with scleritis and 329 control subjects	Evaluation of blood 25(OH)D levels and ophthalmic examinations	Hypovitaminosis D is associated with increased risk of scleritis	[118]
Dry eye syndrome				
Systematic review and meta-analysis	Literature database	Identification of the association between vitamin D level and DES	Significantly lowered serum level of 25(OH)D in DES	[147]
Systematic review and meta-analysis	Literature database	Identification of the association between vitamin D level and DES	Vitamin D deficiency is associated with severity of DES	[148]
Systematic review and meta-analysis	Literature database	Identification of the association between vitamin D level and DES	Vitamin D deficiency may be a risk factor for DES	[149]
Study based on Korea National Health and Nutrition Examination Survey	17,542 adults (7434 men and 10,108 women) aged 19 years	Evaluation of blood 25(OH) levels and clinical examinations	Decreased serum 25(OH)D levels and inadequate sunlight exposure are associated with DES. The vitamin D supplementation may be useful in DES treatment	[150]
Cross-sectional study	47 patients with evaporative DES and 33 control subjects	Evaluation of blood/tear 25(OH) levels and clinical examinations	Significantly lowered tear level of 25(OH)D in DES	[151]
Observational study	34 patients with vitamin D deficiency and 21 control subjects with normal level of vitamin D	Evaluation of blood/tear 25(OH) levels and ophthalmic examinations	Vitamin D deficiency results in the TBUT and Schirmer’s test values	[152]
Single-center, cross-sectional observational study	30 patients with vitamin D deficiency and 30 control subjects with normal level of vitamin D	Evaluation of blood/tear 25(OH) levels and ophthalmic examinations	Vitamin D deficiency is associated with tear hyperosmolarity and tear film dysfunction	[153]
Retrospective observational study	79 patients (22 male and 57 female)	Evaluation of blood 25(OH)D levels and ophthalmic examinations	Tear break-up time and tear secretion were correlated with serum vitamin D levels	[154]
Study based on Korea National Health and Nutrition Examination Survey	16,396 participants aged >19 years	Evaluation of blood 25(OH)D levels and ophthalmic examinations	There is no association between 25(OH)D levels and DES	[155]
Study based on Study Group for Environmental Eye Disease	740 subjects (253 men and 487 women)	Evaluation of blood 25(OH)D levels and ophthalmic examinations	There is no association between 25(OH)D levels and DES	[156]
Observational study	105 (21 men and 84 women) with mean serum 25(OH)D level of 10.52 ± 4.61 ng/mL	Evaluation of blood 25-(OH)D levels and ophthalmic examinations; Intramuscular injection of 200,000 IU cholecalciferol for participants with deficient or insufficient vitamin D level	The supplementation of vitamin D is effective and useful in the treatment of patients with DES	[157]
Retrospective, observational study	116 patients with DES	Evaluation of blood 25-(OH)D levels and ophthalmic examinations	The effect of topical artificial tears and hyaluronate in the therapy of DES is dependent on serum 25(OH)D levels. The cholecalciferol supplementation enhanced the efficacy of topical DES treatment	[158]
Case-control study	64 patients with DES and 51 control subjects	Identification of the association between *VDR* gene polymorphisms and DES susceptibility	Two polymorphisms (Foklrs2228570 and Apal-rs7975232) in the *VDR* gene are 1.72 and 1.66 times more likely in DES-patients than in control, respectively	[22]
Vernal keratoconjunctivitis				
Case-control study	47 patients with VKC, aged between 5 and 12 years, and 63 healthy children	Evaluation of blood 25-(OH)D levels and ophthalmic examinations	Children affected by VKC have lower vitamin D levels. Significant correlation between the disease severity and the level of vitamin D	[161]
Prospective, observational, caseـcontrol study	39 patients with VKC (21 men and 18 women) and 32 health subjects (19 men and 13 women) with the mean age of 18.38 ± 8.83 and 21.6 ± 9.43, respectively	Evaluation of blood 25-(OH)D levels and ophthalmic examinations	Patients with VKC have lower vitamin D levels; No significant reverse correlation between the disease severity and the level of vitamin D	[162]
Prospective, single-centered, observational, case–control study	29 children with VKC and 62 health children	Evaluation of blood 25-(OH)D levels and ophthalmic examinations	Children with VKC should be evaluated for vitamin D deficiency, which might occur secondary to sun avoidance	[163]
Observational study	242 children with VKC	Evaluation of blood 25-(OH)D levels and ophthalmic examinations	Ocular treatment using immunomodulatory eye drops results in an improvement in 25OHD serum levels	[165]
Keratoconus				
Prospective, single-centered, observational case-control study	100 patients with keratoconus and 100 health control subjects	Evaluation of blood 25-(OH)D levels and ophthalmic examinations	Patients with KC had reduced serum vitamin D levels compared to age- and sex-matched healthy controls	[170]
Cross-sectional study	100 patients with keratoconus and 100 control subjects	Evaluation of blood 25-(OH)D levels and ophthalmic examinations	Lower serum 25(OH)D was found in patients with keratoconus compared to the control group	[171]
	55 patients with keratoconus (28 progressive and 27 non-progressive) and 30 age- and sex-matched control subjects	Evaluation of blood vitamin D levels and ophthalmic examinations	Serum vitamin D evaluation in patients with keratocnous at onset and follow-up examinations may help to predict the course of the disease	[172]
Pterygium				
Study based on Korea National Health and Nutrition Examination Survey	19,178 participants aged 30 years	Evaluation of blood 25-(OH)D levels and ophthalmic examinations	Positive association between blood 25(OH)D levels and pterygium	[179]
Prospective study	63 patients with pterygium and 58 control subjects	Evaluation of blood 25-(OH)D levels and ophthalmic examinations	Increased level of blood 25(OH)D in only male subjects with pterygium and in those with more outdoor activity	[180]
Population-based, cross-sectional study based on Korea National Health and Nutrition Examination Survey	12,258 adults (aged ≥ 19 years)	Evaluation of blood 25-(OH)D levels and ophthalmic examinations	Association of daily sun exposure and serum 25(OH)D levels in pterygium	[178]
Case-control study	35 (21 male, 14 female) patients with unilateral pterygium and 25 (18 male, 7 female) healthy controls	Evaluation of blood/tear vitamin D levels and ophthalmic examinations	Tear fluid and serum vitamin D concentrations do not have a role in pterygium pathogenesis	[181]
Cross-sectional study	50 patients with pterygium and 50 control subjects	Identification of the *VDR* gene expression in pterygium; Identification of the *VDR* gene polymorphisms and DES susceptibility	*VDR* expression is increased in thepterygium tissue compared to the adjacent healthy tissue. No significant difference in *BsmI*, *FokI* and *TaqI* polymorphisms in comparison to the control	[182]
Thyroid eye disease				
Retrospective chart review	35 patients with TED	Evaluation of blood 25-(OH)D levels and clinical examinations	20% prevalence of vitamin D deficiency in TED	[188]
Retrospective case-control study	89 TED patients and 89 Graves disease patients without TED, and 2 healthy control groups matched 4:1 to the cases; 356 health control patients matched to the TED group and 356 health control patients matched to the Graves disease	Evaluation of blood 25-(OH)D levels and clinical examinations	Low serum vitamin D is associated with TED diagnosis	[189]
Case-control study	108 patients with thyroid-associated orbitopathy and 130 health control subjects (Caucasian Polish origin)	Identification of the *VDR* gene polymorphisms and thyroid-associated orbitopathy susceptibility	C allele of *rs2228570 VDR* gene polymorphism may contribute to the development of thyroid-associated orbitopathy	[190]
Benign essential blepharospasm				
Prospective study	50 patients with BEB and 22 health subjects	Evaluation of blood 25-(OH)D levels and clinical examinations	Serum vitamin D levels showed a moderate negative correlation with disease severity	[194]
Retrospective case-control study	20 patients with BEB and 20 age- and gender-matched health subjects	Evaluation of blood 25-(OH)D levels and clinical examinations	Strong negative correlation between disease severity and reduced 25(OH) vitamin D in patients with BEB	[196]

## Abbreviations

AMD, age-related macular degeneration; DES, dry eye syndrome; DR, diabetic retinopathy; IOP, intraocular pressure; ON, optic neuritis; RVO, retinal vein occlusion; UM, uveal melanoma; VDR, vitamin D receptor; VKC, vernal keratoconjunctivitis; VKHD, Vogt-Koyanagi-Harada disease.
